# Supplementary material for: The Evolution and Characterization of the RNA Interference Pathways in Lophotrochozoa
Source: Genome Biol Evol. 2024 May 7;16(5):evae098. doi: 10.1093/gbe/evae098 (PMC11114477; doi:10.1093/gbe/evae098)
Supplement: evae098_Supplementary_Data [file evae098_supplementary_data.zip › Supplementary_materials.zip!/selected_miRNAs/NoGe_selected_miRNAs/known_miRNA_families_pdf_files/NMRB01002521.1_Notospermus_geniculatus_strain_Ushimado_scaffold2521,_whole_genome_shotgun_sequence_38687.pdf]

| | | |
|---|---|---|
| Provisional ID | : | NMRB01002521.1_Notospermus_geniculatus_strain_Ushimado_scaffold2521,_whole_genome_shotgun_sequence_38687 |
| Score total | : | 7330434.3 |
| Score for star read(s) | : | 3.9 |
| Score for read counts | : | 7330425.8 |
| Score for mfe | : | 1.5 |
| Score for randfold | : | 0 |
| Score for cons. seed | : | 3 |
| Total read count | : | 14378326 |
| Mature read count | : | 14378019 |
| Loop read count | : | 8 |
| Star read count | : | 299 |

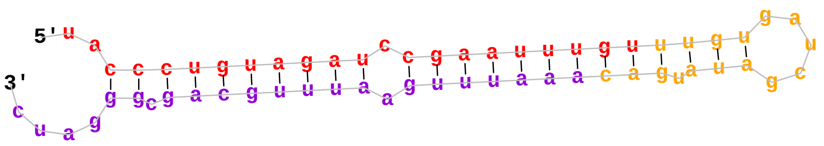


Genome coordinates: NMRB01002521.1 85025-85086

pre-miRNA sequence:
UACCCUGUAGAUCCGAAUUUGUUACCCUGUAGAUCCGAAUUUGUUUGUGAUCGAUAUGACAAAUUUGAAUUUGCAGCGGGAUC

Mature sequence: UACCCUGUAGAUCCGAAUUUGU

Aligned mature sequence of the *N. geniculatus* pre-miRNA with the top 5 hits from the BLAST search against the pre-miRNA mirGeneDB set:

```
                                   **********************
Nge_Mir-10-P1-v2                   uacccuguagauccgaauuugu
Aae-Mir-10-P1-v2_5p                uacccuguagauccgaauuugu
Bge-Mir-10-P1-v2_5p                uacccuguagauccgaauuugu
Hme-Mir-10-P1-v2_5p                uacccuguagauccgaauuugu
Lpo-Mir-10-P1k-v2_5p               uacccuguagauccgaauuugu
Tca-Mir-10-P1-v2_5p                uacccuguagauccgaauuugu
```
